# Myocardial Bridge and Acute Plaque Rupture

**DOI:** 10.1177/2324709616680227

**Published:** 2016-12-01

**Authors:** Leor Perl, David Daniels, Jonathan Schwartz, Shige Tanaka, Alan Yeung, Jennifer A. Tremmel, Ingela Schnittger

**Affiliations:** 1 Stanford University Medical Center, Stanford, CA, USA; 2 Palo Alto Medical Foundation, Burlingame, CA, USA

**Keywords:** myocardial bridge, acute coronary syndrome, intravascular ultrasound

## Abstract

A myocardial bridge (MB) is a common anatomic variant, most frequently located in the left anterior descending coronary artery, where a portion of the coronary artery is covered by myocardium. Importantly, MBs are known to result in a proximal atherosclerotic lesion. It has recently been postulated that these lesions predispose patients to acute coronary events, even in cases of otherwise low-risk patients. One such mechanism may involve acute plaque rupture. In this article, we report 2 cases of patients with MBs who presented with acute coronary syndromes despite having low cardiovascular risk. Their presentation was life-risking and both were treated urgently and studied with coronary angiographies and intravascular ultrasound. This latter modality confirmed a rupture of an atherosclerotic plaque proximal to the MB as a likely cause of the acute events. These cases, of unexplained acute coronary syndrome in low-risk patients, raise the question of alternative processes leading to the event and the role MB play as an underlying cause of ruptured plaques. In some cases, an active investigation for this entity may be warranted, due to the prognostic implications of the different therapeutic modalities, should an MB be discovered.

## Introduction

Myocardial bridges (MBs) are common anatomic variants,^1,2^ where a portion of the epicardial coronary artery is covered by myocardial tissue. This phenomenon is most frequent in the left anterior descending coronary artery (LAD) and is known to predispose to ischemia due to both a mechanical compression of the vessel at the site of the bridge and as a result of an atherosclerotic lesion, invariably developing proximal to it.^3-5^ The atherosclerotic plaque is thought to have been formed by mechanical causes, due to blood flow alteration from systolic compression of an MB, leading to low shear stress proximal to it, evoking lipid transfer augmentation under the endothelium.^1,6^ This process was recently discovered to be particularly prone to rupture, thus increasing the risk of acute coronary events at an early age.^5^ However, this potentially catastrophic consequence of an MB has yet to be fully addressed, especially when considered in young and low-risk patients presenting with an acute coronary syndrome (ACS). Two such examples are described below.

## Case Descriptions

**Figure 1.**
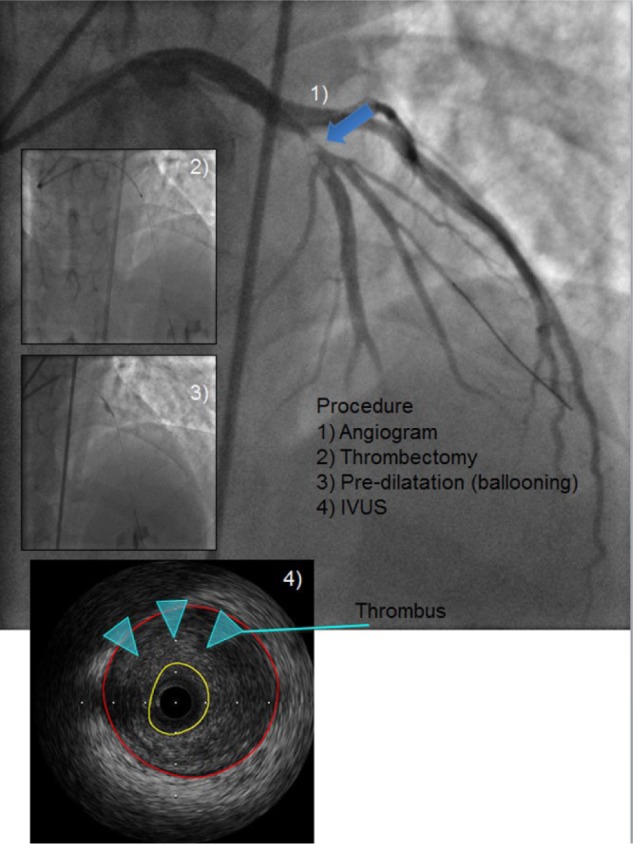
Coronary angiogram (1) showing a hazy lesion in the proximal LAD (single blue arrow). Following thrombectomy (2) and predilatation with balloon angioplasty (3), IVUS demonstrated a thrombus (turquoise arrow heads) in the culprit lesion (4). LAD, left anterior descending artery; IVUS, intravascular ultrasound.

**Figure 2.**
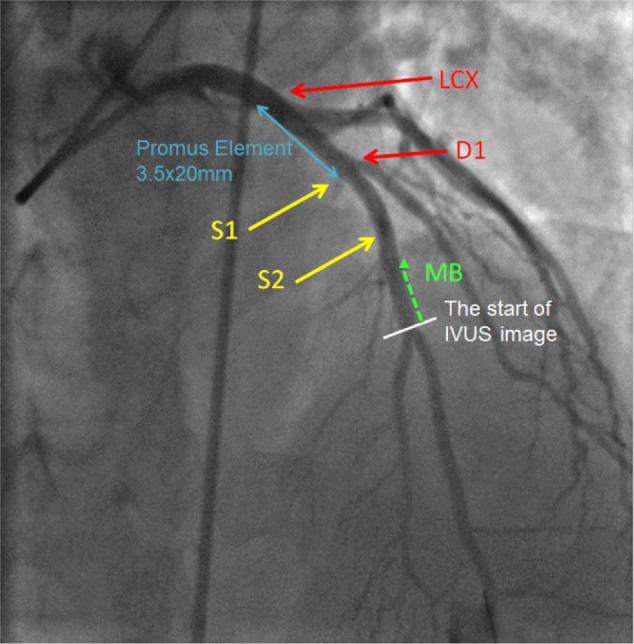
The final angiogram showing a good result of stenting of the proximal LAD, before the take-off of the first diagonal branch. LAD, left anterior descending artery; LCX, left circumflex artery; D1, first diagonal branch; S1, first septal perforator artery; S2, second septal perforator artery; MB, myocardial bridge.

**Figure 3.**
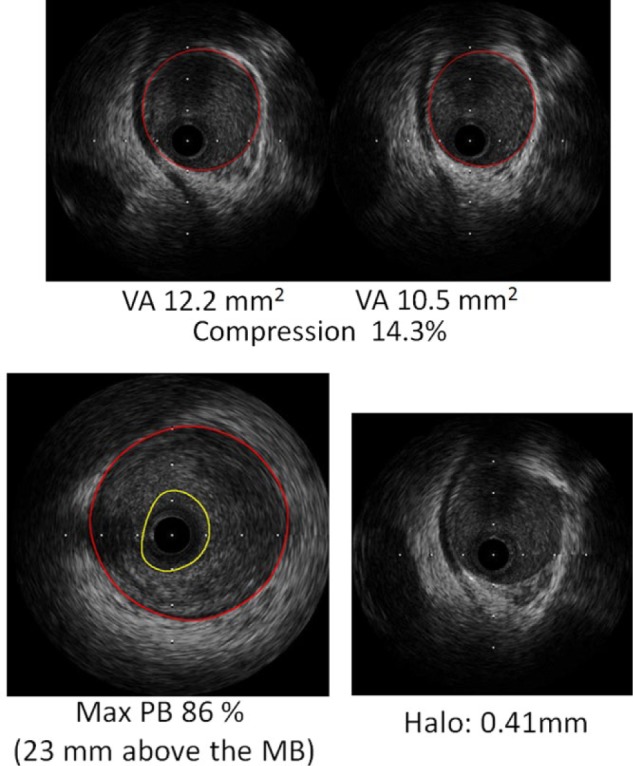
Top panel: IVUS still frames demonstrating cross-sectional images of vessel area (VA) from within the MB segment from end-diastole and end-systole resulting in an arterial compression of 14.3 %. Bottom panel: The maximum plaque burden (PB), the difference in cross-sectional area between the red circle (the external elastic membrane) and yellow circle (lumen intima border) was 86%. The MB thickness (halo) was 0.41 mm. MB, myocardial bridge; IVUS, intravascular ultrasound.

A 47-year-old man with no significant past medical history, and no risk factors for atherosclerosis, presented to us with an anterior ST-elevation myocardial infarction. He reported an event of some minor chest pressure 2 to 3 weeks prior to admission. On the morning of admission, he developed severe chest pain accompanied by diaphoresis and dyspnea. The patient was taken urgently to the catheterization laboratory. During transport to the laboratory he developed ventricular fibrillation, from which he was successfully resuscitated with a single defibrillation. He then had an angiogram, which demonstrated a 100% thrombotic occlusion of the proximal LAD, without any other significant lesions. A mechanical thrombectomy was performed, followed by balloon angioplasty and a drug-eluting stent implantation, with excellent angiographic results. An intravascular ultrasound (IVUS) performed during the procedure identified a MB 23 mm distal to the point of maximal plaque burden. The MB was greater than 12-mm long, had a systolic compression of 14.3%, and an MB thickness (halo) of 0.41 mm (Figures 1-3). Clinical outcome was good; the patient remained stable during the hospitalization and is asymptomatic since then.

**Figure 4.**
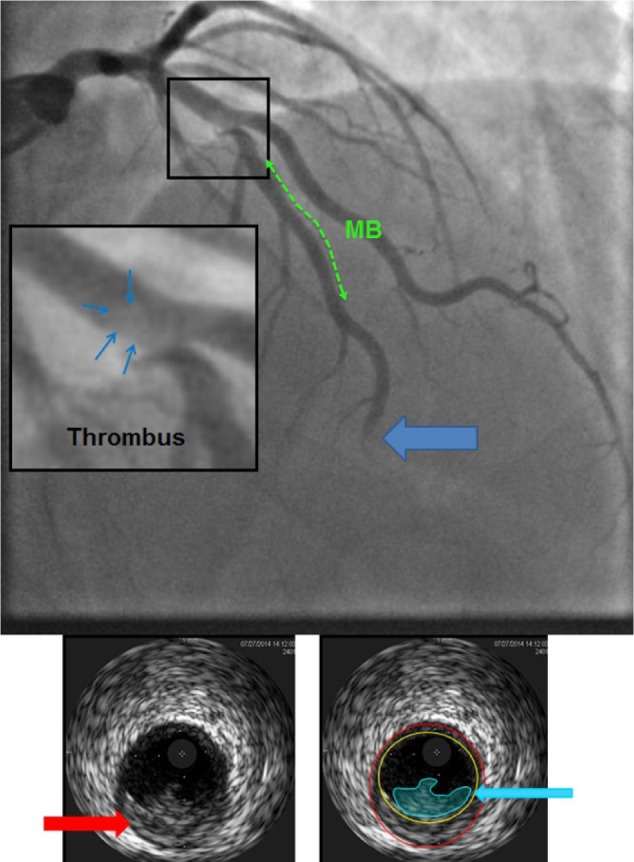
Coronary angiogram showing a hazy lesion in the proximal LAD, consistent with an acute thrombus formation. The green area shows the MB segment. The large blue arrow points to the total occlusion of the mid to distal LAD from distal embolization. On the left: a magnification of the hazy area in the LAD, consistent with a thrombus. Bottom images: the left picture shows an IVUS image of the ruptured plaque (red arrow) and to the right the thrombus on top of the plaque (blue arrow). MB, myocardial bridge; LAD, left anterior descending artery; IVUS, intravascular ultrasound.

**Figure 5.**
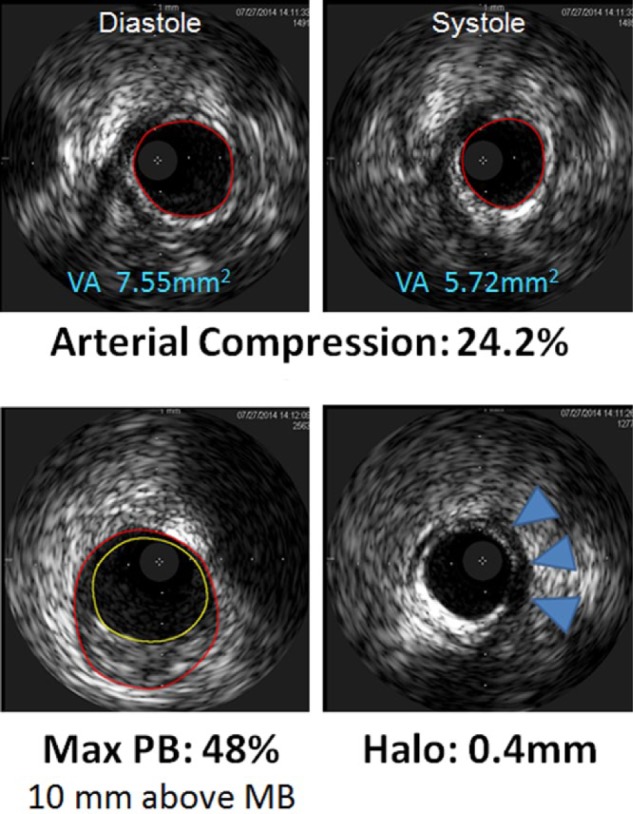
Top panel: IVUS still frames demonstrating cross-sectional images of vessel area from within the MB resulting in an arterial compression of 24.2%. Bottom panel: The maximum plaque burden (PB) was 48%. The halo (MB thickness), blue arrows, was 0.4 mm. MB, myocardial bridge; VA, vessel area; IVUS, intravascular ultrasound.

A 41-year-old man, a visitor from Europe and an athlete, with borderline hyperlipidemia and no other past medical history, had completed the San Francisco Marathon on the morning of the index event. He had vomited twice shortly after the race, and then returned to his hotel. Two hours later, he developed substernal chest pain, lasting approximately 30 minutes, accompanied by shortness of breath. Learning about the possible cause of the symptoms from the World Wide Web, he decided to contact emergency medical services. He was brought in to the emergency department, where an EKG (electrocardiogram) showed ST elevations in the anterior and inferior leads, with sinus bradycardia. Coronary angiography was urgently performed, revealing an intermediate lesion in the proximal LAD, as well as a total cut-off of the distal wraparound LAD, likely due to the proximal plaque rupture and distal embolization. The rest of the coronary tree did not demonstrate angiographic evidence of disease. Following mechanical thrombectomy of the embolized thrombus and restoration of TIMI 3 flow, IVUS of the artery demonstrated a proximal plaque rupture, as well as a mid-LAD MB. The bridge was located 10 mm distal to the plaque, and measured 14 mm in length, had a systolic arterial compression of 24.2%, and had a halo of 0.4 mm (Figures 4 and 5). Left ventriculography showed a normal ejection fraction, with anterior and apical hypokinesis. A drug-eluting stent was implanted successfully in the proximal lesion, without complications. The patient later suffered from transient nonsustained ventricular tachycardia, but otherwise was asymptomatic, and had remained so after his discharge. He returned to his home country later that month.

## Discussion

Myocardial bridges were first described in 1737, as an anatomic course of an epicardial vessel through myocardial fibers,^7^ but it was not until the emergence of coronary angiography that Porstmann and Iwig^8^ were able to show the functional characteristics of this phenomenon. The prevalence of an MB varies considerably, depending on the diagnostic method. On angiography, rates of MBs have been reported to be 0.5% to 5%, depending on the different patient population,^6^ whereas in autopsy studies, the rates may be as high as 86%,^9^ and on computed tomography, an MB will be identified at a prevalence of 6% to 15%, in patients with suspected coronary artery disease.^10,11^ Every year, it is estimated that approximately 635 000 suffer a new myocardial infarction in the United States alone, and close to 400 000 die of coronary heart disease.^12^ Most of these events are associated with a vulnerable plaque, one that is not flow-limiting until it is ruptured or eroded.^13^ Traditional cardiac risk factors are generally felt to be the cause of plaque development, but in those with few or no cardiac risk factors, other etiologies should be considered. An MB originally was thought to be regarded as a common, benign, anatomic variant rather than a congenital anomaly.^6^ However, more recent data suggest otherwise. We have shown a correlation between the degree of systolic compression of the MB by IVUS and the degree of atherosclerotic plaque developing proximal to an MB.^14^ The degree of compression of the MB was a stronger predictor of maximum plaque burden proximal to the bridge than MB length, depth, or location of the MB. This was particularly true for young patients with low coronary risk. These plaques are infrequently appreciated by contrast coronary angiography because of eccentric remodeling of the vessel, but they are invariably seen by IVUS, generally up to 20 mm proximal to the MB entrance^14^ and may be one of the pathologic mechanism for myocardial infarction associated with myocardial bridging. It has also been demonstrated that the tunneled segment is essentially spared from atherosclerosis.^15,16^ The reason for the preferential plaque build-up proximal to MB is likely multifactorial; the distribution of wall shear stress is lower proximal to MB than inside MB, which has been postulated to predispose to enhanced lipid transfer across the endothelium.^17^ On the contrary, the wall shear stress is high inside the MB,^18^ sparing this segment from any significant plaque buildup.^19^ Another potential mechanism for proximal plaque formation includes abnormal blood flow profiles at the proximal segment, because of reversal of flow from the contracting MB, leading to potential collision with ante grade coronary flow. This may create turbulence and trauma to the endothelial lining of the vessel at that location.^20^ Scanning electron microscopy studies on endothelial cells lining the LAD showed that these cells are polygonal and flat in the LAD intima proximal to the MB entrance, but become spindle and engorged and align in the direction of blood flow beneath the MB.^21^ Importantly, Ishikawa et al^5^ have shown that MBs contribute to unique intimal lesions in the proximal LAD, lesions that induce plaque rupture, resulting in clinical events at a young age.

Myocardial bridges are congenital anomalies. Our own experience in clinical practice points to a hereditary relationship as we have encountered many families with this condition. However, the mode of inheritance has not been elucidated thus far. As a potential biomarker for MB, one group of investigators has reported 5 micro-RNAs that showed the ability to distinguish MB patients from controls.^22^

This article describes 2 cases of young patients presenting with life-threatening ACSs, with likely a ruptured plaque as the etiology for the acute pathologic event and a MB distal to the plaque. The atherosclerotic burden in the remainder of the coronary tree of these patients remained low. In cases of relatively young patients and without significant risk factors, presenting with ACSs with an ulcerated plaque, an active investigation for the presence of an MB should be considered, as it may play a larger role in these events than we currently appreciate. Due to the fact that coronary angiography is inadequate at demonstrating an MB, IVUS should be considered as part of the investigation. It is especially important to know of the presence of an MB if there is intention to treat the LAD percutaneously, in which case an MB is associated with worse clinical outcomes,^23^ particularly if the stent enters the MB.^24^ Other modalities of therapy could also be considered, if the clinical situation permits, including surgical myotomy for those with chronic angina who have failed medical therapy. We have recently shown a significant improvement in chest pain and in quality of life in a series of 50 patients who underwent successful surgical unroofing of an MB.^25^ In the case of a significant flow-limiting plaque, left internal mammary artery grafting to the LAD^26-28^ should be considered, as well, in addition to unroofing of the entire length of the bridged arterial segment.

## Conclusion

An MB is a common anatomic variant of the coronary arteries, where a portion of the artery is covered by a myocardial band. It has recently been shown to predispose to the development of an atherosclerotic plaque proximal to the bridge. It is yet unknown as to what percentage of unexplained ACS cases may be attributed to the formation of coronary plaques proximal to an MB, with subsequent plaque rupture. The cases presented here illustrate the importance of this phenomenon, especially in younger patients, and the need to consider this risk factor, indeed a congenital one, in the investigation and management of patients with an MB.
